# Knowledge towards Strabismus and Associated Factors among Adults in Gondar Town, Northwest Ethiopia

**DOI:** 10.1155/2020/3639273

**Published:** 2020-04-24

**Authors:** Aragaw kegne Assaye, Melkamu Temeselew Tegegn, Natnael Lakachew Assefa, Betelhem Temesgen Yibekal

**Affiliations:** Department of Optometry, School of Medicine, College of Medicine and Health Science, University of Gondar, Gondar town, Ethiopia

## Abstract

**Introduction:**

Strabismus/squint is an ocular misalignment in which the eyes are not properly aligned with each other. It is an avoidable cause of blindness and has a global prevalence which ranges from 2% to 6%. Knowledge of eye diseases is important in encouraging people to seek early treatment, which further helps in reducing the burden of visual impairment. Studies in Ethiopia showed that the level of good knowledge was 37%. There is a lack of information regarding knowledge and related factors of strabismus in the study area and limited in Ethiopia at large.

**Objective:**

The aim of this study was to asses knowledge about strabismus and associated factors among adults in Gondar town, Northwest Ethiopia.

**Methods:**

Community-based cross-sectional study was conducted using a pretested structured questionnaire through face to face interview from April 17 to May 01, 2019. Using multistage random sampling, 553 participants were included. Data from the entire questionnaire were coded, entered into Epi info version 7, and exported to SPSS version 20 for processing and analysis. Binary logistic regression was fitted, and variables with *P* value <0.05 in the multivariable logistic regression were considered as statistically significant.

**Results:**

A total of 553 adults with a response rate of 93.25% participated in the study. The median age was 33 (IQR = 14) years. Among the participants, 52.3% (95% CI: 47.9–56.4) had good knowledge towards strabismus. Being a student (AOR = 2.15, (95% CI: 1.11–4.13) was positively associated, while monthly income >5000 birrs (AOR = 0.44, 95% CI: 0.26–0.76) was negatively associated with good knowledge about strabismus.

**Conclusion:**

Almost half of the participants had good knowledge. Occupation and monthly income had a significant association with knowledge about strabismus. Since the source of information (TV/radio and Internet) was extremely important for the reliability and the level of knowledge, it is better to have sufficient media coverage.

## 1. Introduction

Eyes are well aligned, so the foveae (corresponding retinal focus point) are aimed at the same visual target; this is termed as orthophoria. Due to different factors, eyes are deviated from its normal position, alternatively termed as strabismus, squint, oblique eye, or heterotropia. These terms come from the fact that strabismic patients often squint one eye to block out one of the two images that they see. Therefore, strabismus is an ocular misalignment in a different direction of gaze, or the eyes are not properly aligned with each other, whether caused by abnormalities in binocular vision or by anomalies of neuromuscular control of ocular motility [1–4].

Globally, the prevalence ranges from 2 to 6% [5–7]. The prevalence of strabismus was found to be 3.3% in whites, 2.1% in African American children [8], 1–4% in African [9–12], 2.4% in UK, [7] 2% in south East Iran [13], 3.1% in Sweden, [7] 5% in Saudi Arabia [14], 2.8% in Australia [7], 5.9% in Tanzania [10], 2.8% in Sudan [11], and 1.53% in Ethiopia [12].

Strabismus can develop at any age but usually develops during childhood, before 6 years of age; the peak age of onset is around 3 years. Strabismus in adulthood frequently occurs secondary to either systemic disease or mechanical damage such as trauma or brain tumor [15, 16].

The risk factors of strabismus are uncorrected refractive error, maternal health, premature birth, low birth weight, developmental delay, syndromes (a group of disease), genetic factors, systemic illnesses, and mechanical agents [16, 17].

Strabismus can be managed with eyeglasses, prisms, surgery, eye exercises, and medicines [16]. If left untreated, it results in abnormal fixation, double vision, abnormal head posture, and lazy eye (amblyopia). Untreated strabismus can also cause psychosocial effects, low performance in school, loss of confidence and self-esteem, and inability in employments and social stigma [18, 19].

In order to tackle such problems, parents/guardians should have good knowledge of the nature of strabismus/squint because most of them do not know the appropriate and effectiveness of the treatment [9, 20].

Studies conducted in different regions of the world showed that there is a knowledge gap about strabismus. In India, 94.7% of participants had knew the consequence of strabismus [21], in Jeddah, 75% of participants knew etiologies [22], in Nigeria, 50% of participants did not know strabismus [23], and in Ethiopia, only 37.2% knew the cause of strabismus [9].

There were studies conducted on the prevalence and psychological effects of strabismus. Knowledge among adults towards strabismus is critical for children's health because it is important for early prevention of visual impairments, to decrease psychological trauma, to lessen the economic burden, to avoid social stigma, and to minimize poor school performance and unemployment. Despite all these uses, little is known about strabismus in Ethiopia in general and in the study area specifically. So, this study can give baseline information on strabismus and associated factors.

## 2. Materials and Methods

### 2.1. Study Design and Period

A community-based cross-sectional study was conducted from April 17 to May 01, 2019.

### 2.2. Study Setting

The study was conducted in Gondar town. Gondar town is located in Northwest Ethiopia. It is located 748 km from the capital, Addis Ababa, and 182 km from Bahir Dar, the capital of Amhara National Regional State. According to Gondar town statistics agency, 2016/17 projection has a population size of 351,675, out of which 168,993 are males and 182,682 are females. According to North Gondar information and statistics agency, the town is subdivided into 6 subcities and 24 city kebeles, holding approximately 53,725 households. The health coverage was 30% in 2007 [24]. There is one government hospital, University of Gondar Tertiary Eye Care and Training Centre (UoGTETC), which provides different specialty eye care services and give training for ophthalmologists, optometrists, and ophthalmic nurses. In the town, there are three private eye care clinics.

### 2.3. Sample Size and Sampling Technique

A total of 593 samples was determined using single population proportion formula by assuming 95% confidence level, 5% margin of error, 10% nonresponse rate, design effect 1.5, and the proportion of good knowledge from a similar study conducted in central Ethiopia, Cheha district (37.2%) [9].

A multistage sampling technique was employed. Six kebeles were selected from 24 kebeles by a simple random sampling technique. In selected kebeles, there were 189, 675 adult populations and 12,952 households. Proportion allocation was used to determine the sample size in each kebele. Households in each kebele were selected by a systematic random sampling method using a sampling fraction (*K*) of 22. One adult was selected using a lottery method from one household if there were two or more adults per household to obtain a final sample. All adults aged ≥18 years had an equal likely chance to participate in this survey. Nevertheless, those adults who had a mental illness and unable to speak were excluded.

### 2.4. Data Collection Tool and Procedure

Data were collected through face-to-face interviews, using a pretested structured hard copy questionnaire. It has questions for sociodemographic characteristics, past ocular history-related factors, source of information socioeconomic factors, and knowledge-related questions such as definition, risk factor, treatment, and consequence of strabismus after reviewing the related literature.

The original questionnaire was translated from English to Amharic version and then translated back to English by two independent local language translators to maintain its consistency and accuracy. The interview was conducted by 8 BSc nurses.

### 2.5. Operational Definitions

#### 2.5.1. Knowledge

The knowledge of respondents was assessed through different dimensions of strabismus, including definitions, causes, treatment, and consequences that scale up 28 points. Each correct response scored as 1 and the incorrect one was coded as 0 (zero).

*(1) Good Knowledge*. Respondents who answered greater than or equal to the median score of knowledge-related questions were said to have good knowledge, otherwise, poor knowledge.

*(2) Adults*. Individuals with age of 18 years and older [25].

*(3) Role in the Community*. Those participants having responsibility and in position in a local area such as kebele leader and religious leader.

### 2.6. Data Quality Control

The training was given for data collectors before the beginning of data collection. The questionnaire was pretested on 5% of the sample size outside the study area. Also, data clean up, checking for data completeness, outliers, and missing values, and supervision were carried out.

### 2.7. Data Processing and Analysis

Data from the entire questionnaire were coded, entered into Epi info version 7, and exported to SPSS version 20 for processing and analysis. Frequency, statistical summary, and cross-tabulations were used for the descriptive analysis of the entered data. Binary logistic regression was used to identify factors associated with knowledge about strabismus. All the variables were entered into multivariable logistic regression, and *P* value <0.05 was taken as statistically significant. The model fitness was checked according to Hosmer and Lemeshow goodness of fit. Finally, the analyzed result was presented using tables and charts.

### 2.8. Ethical Consideration

Ethical clearance was obtained from University of Gondar College of Medicine and Health Sciences, School of Medicine, ethical review committee. After informing about the objective of the study, verbal informed consent was obtained from each study participant. The questionnaires did not require the identifiers of the participants. Confidentiality of the information obtained was assured and maintained anonymous. Participants who had strabismus or any vision threatening eye problems were strongly advised to visit Gondar University Tertiary Eye care and Training Center Hospital. The collected data were securely locked.

## 3. Results

### 3.1. Sociodemographic Characteristics of the Study Participants

A total of 553 among 593 participants who were living in Gondar town took part in the study with a response rate of 93.25%.

Of the total participants, 53.7% (297) were males. The median age of the study participants was 33 (IQR = 14) years. Most (81.4% (450)) of the study participants were orthodox Christian. Half (50.7% (305)) of the participants were employed. Of the total participants, 39.8% (308) were married. The median monthly income was 3800 ETB (IQR = 2500, *Q*1 = 2500, *Q*3 = 5000). More than half (65.3% (393)) of the participants had an education level of college and above (Table 1).

### 3.2. Proportion of Knowledge towards Strabismus among Study Participants

Out of 553 study participants, 52.30% (289) (95% CI: 47.9–56.4) had good knowledge about strabismus.

**Figure 1:**
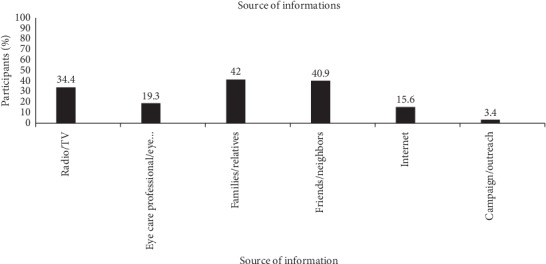
Source of information among study participants in Gondar town, Northwest Ethiopia, 2019.

Among study participants who had heard about strabismus, 15 (2.7%) did not know what strabismus is. Of the study participants, the most perceived definition, cause, treatments, and consequences of strabismus were as follows: two eyes not coordinated, 72.0% (398), exposure to sunlight/lamp, 66.4% (367), surgery, 57.1% (316), and poor cosmoses, 82.1% (347), respectively (Table 2). The most frequently mentioned source of information was from families/relatives, 42% (232), followed by radio/television, 37.4% (207), and the least one was from Internet, 15.65% (86) (Figure 1).

### 3.3. Factors Associated with Level of Knowledge towards Strabismus

The result of multivariable logistic regression showed that occupations and monthly income were significantly associated with knowledge about strabismus.

Those participants who were students were 2.15 times more likely to have good knowledge than those who were employed (AOR = 2.15, (95% CI: 1.11–4.13). Participants who earn a monthly income of >5000 birr were 54% less likely to have good knowledge about strabismus as compared to those who get <2500 birr (AOR = 0.48, (95% CI: 0.26–0.46)) (Table 3).

## 4. Discussion

This is perhaps the first study in the Amhara region and the second in Ethiopia to estimate the level of knowledge among adults towards strabismus in Gondar town, Northwest Ethiopia. In this study, 553 participants were included, of whom 52.3% (95% CI: 47.9–56.4) of participants had good knowledge regarding strabismus. This result was higher than a study conducted in Cheha district of Central Ethiopia (37%) [9]. This may be due to the difference in sociodemographic characteristics of the participants since 51.7% of participants were illiterate in Cheha study but only 9% of this study participants were illiterate [9]. On the contrary, the current study finding was lower than a study conducted in Kenya (69.60%) [26]. This discrepancy may be due to the difference in study population, which was only health profession for the study in Kenya and all adults in the community in this study.

Regarding different dimensions of strabismus, proportions of participants' knowledge about the alternative definitions (43–72%), causes (15–30%), treatments (39–57%), and consequences (29–82%) were higher than in a study conducted in Cheha district, central Ethiopia, showing knowledge about the causes (4.3–37.2%), treatments (32%), and consequences (3.8–43%). This study result is also higher than a study conducted in Nigeria (46%) [23]. This may be due to the difference in the study population who were agrarian and rural dwellers, which made them less aware of strabismus. However, this finding was lower than a study conducted in Jeddah (60–79%), India (94.7%), and Saudi (66.7%) [21, 22, 27]. This is due to the difference in the study setting since these studies were hospital-based.

Participants who had monthly income >5000 birr were 54% less likely to have good knowledge as compared to those who had <2500 birr. This may be due to those having a high amount of monthly income such as merchants (46.42%) who might work for long hours, which may hinder them to access available information. Participants who were students were 2.15 times more likely to have good knowledge than those who were government employees. This may be due to students who were observed in this study who used a different source of information such as the Internet and mass media, which help them to get more knowledge about strabismus.

Source of information was an extremely important dimension that should be considered while designing relevant preventive or screening programs. Gaining knowledge from the relative (38.5%) or friends (37.5%) was the predominant source of information about strabismus. Interestingly, the Internet and radio/TV constituted a considerable proportion of reliable knowledge sources. This may be due to the messages delivered through radio/TV in a coherent and scientific manner. This was typically consistent with another study in India, Saudi, and Jeddah [21, 22, 27], in which both radio/TV and the Internet were the reliable sources of knowledge than relatives/friends.

Regarding factors, some studies did not analyze the association between all presumed factors and the overall knowledge level, rather to different dimensions of knowledge about strabismus. For example, in Cheha district, Central Ethiopia [9], age and educational status were significantly associated with the knowledge of treatment but not to the causes of strabismus. This makes a comparison of this study results with other studies difficult since the association between the overall knowledge and different factors is considered in this study.

## 5. Conclusion

The overall knowledge score of the study participants was moderate. More than half of the study participants had good knowledge about strabismus. It was found that being a student and having high monthly income had a statistically significant association with knowledge about strabismus. Since the type and source of information were extremely important for reliability and the level of knowledge, it is better to improve the availability of media coverage such as radio, TV, and Internet.

## Figures and Tables

**Table 1:** Sociodemographic characteristics of study participants in Gondar town, Northwest Ethiopia, 2019.

Variables	Frequency (*n*)	Percentage (%)
*Age*		
18–28	175	31.6
29–39	194	35.1
40–49	117	21.2
50–59	42	7.6
>60	25	4.5

*Sex*		
Male	297	53.7
Female	256	46.3

*Marital status*		
Married	308	55.7
Single	220	39.8
Widowed	13	2.4
Divorced	12	2.2

*Religion*		
Orthodox	450	81.4
Muslims	72	13.0
Protestants	31	5.6

*Monthly income*		
500–2500	141	25.5
2501–3800	138	25.0
3801–5000	140	25.3
>5000	134	24.2

*Education level* ^*∗∗*^		
No formal education	50	9.0
Primary school	17	3.1
Secondary school	119	21.5
College and above	367	66.4

*Occupations* ^*∗∗*^		
Employed	290	52.4
Housewife	57	10.3
Students	79	14.3
Merchants	84	15.2
Unemployed	22	4.0
Others^*∗∗*^	21	3.8

*Role in the community*		
Had role	35	6.3
No role	518	93.7

Others^*∗∗*^ included farmers and retired people. Occupations^*∗∗*^ included those from Jeddah [22] and Ethiopia [9]. Education level^*∗∗*^ included those from Nigeria [23].

**Table 2:** Participants response regarding different dimensions of strabismus (*n* = 553).

Different dimensions of strabismus	Response of participants			
	Correct response		Incorrect response	
	Frequency (*n*)	Percentage (%)	Frequency (*n*)	Percentage (%)
*Definitions*				
One eye misalignment	240	43.4	313	56.6
Two eye misalignment	268	48.5	285	51.5
Laziness of an eye	312	56.4	241	43.6
Abnormal eye movements	176	31.8	377	68.2
Two eyes not coordinated	399	72.2	154	27.8
Optic nerve degenerations	120	21.7	433	78.3

*Causes/risk factors*				
Heredity	88	15.9	465	84.1
Trauma	182	32.9	371	67.1
Other eye disease	170	30.7	383	69.3
Cataract	93	16.8	460	83.2
Nutritional deficiency	285	51.5	268	49.5
Exposure to lamp/light	112	20.3	441	79.7
Prematurity	28	5.1	525	94.9
Fever during infancy	306	55.3	245	44.7
Redness	373	67.5	180	32.5
Beliefs	424	76.7	129	23.3

*Treatment*				
Eye glasses	219	39.6	334	60.4
Surgery	316	57.1	237	42.9
Eye muscle exercise	95	17.2	458	82.8
Patching better eye	249	45	304	55
Resolves by its self	393	71.1	160	28.9
Eye drops	299	54.1	254	45.9

*Consequences*				
Amblyopia	349	62.9	205	37.1
Poor in school performance	217	39.2	336	60.8
Social stigma	225	46.1	298	53.9
Poor cosmoses	454	82.1	99	17.9
Self-depression	320	57.9	233	42.1
Dependency	161	20.1	392	79.9

**Table 3:** Factors associated with knowledge towards strabismus among adults in Gondar town, Northwest Ethiopia, 2019.

Variables	Knowledge level		COR (95% CI)	AOR (95% CI)
	Good	Poor		
*Age*				
18–28	90	85	1	1
29–39	109	85	1.21 (0.80, 1.82)	1.56 (0.94, 2.56)
40–49	56	61	0.87 (0.54, 1.38)	1.27 (0.69, 2.36)
50–59	20	22	0.86 (0.44, 1.68)	1.27 (0.56, 2.88)
>60	14	11	1.20 (0.52, 2.79)	1.98 (0.63, 6.17)

*Gender*				
Male	151	146	1	1
Female	138	118	1.13 (0.81, 1.58)	1.13 (0.76, 1.68)

*Marital status*				
Married	161	147	1	1
Single	118	102	1.06 (0.75, 1.49)	0.82 (0.52, 1.28)
Widowed	5	8	0.57 (0.18, 1.78)	0.42 (0.12, 1.44)
Divorced	5	7	0.65 (0.20, 2.10)	0.63 (0.19, 2.12)

*Income*				
500–2500	80	61	1	1
2501–3800	77	61	0.96 (0.60, 1.54)	0.95 (0.57, 1.57)
3801–5000	81	59	1.05 (0.65, 0.68)	0.98 (0.59, 1.62)
>5000	51	83	0.47 (0.29, 0.76)	0.44 (0.26, 0.76)^*∗∗*^

*Education level*				
No formal education	26	24	1	1
Primary school	9	8	1.04 (0.34, 3.13)	1.05 (0.32, 3.42)
Secondary school	59	60	0.91 (0.47, 1.76)	0.89 (0.40, 1.95)
College/university	195	172	1.05 (0.56, 1.89)	1.20 (0.55, 2.62)

*Occupations * ^*∗∗*^				
Employee	146	144	1	1
Merchant	42	42	0.99 (0.61, 1.60)	1.35 (0.76, 2.40)
Student	49	30	1.61 (0.97, 2.68)	2.15 (1.11, 4.13)^*∗∗*^
Housewife	28	29	0.95 (0.54, 1.68)	0.98 (0.46, 2.08)
Unemployed	12	10	1.18 (0.50, 2.83)	1.41 (0.54, 3.66)
Others	12	9	1.31 (0.54, 3.22)	1.38 (0.46, 4.10)

*Role in the community*				
Had role	16	19	0.76 (0.38, 1.50)	0.87 (0.42, 1.81)
No role	273	245	1	1

*Health insurance*				
No	267	246	1	1
Yes	18	22	1.13 (0.59, 2.15)	1.01 (0.51, 2.02)

*Previous eye examinations*				
No	199	176	1	1
Yes	90	88	0.90 (0.63, 1.29)	0.91 (0.61, 1.35)

*Eye health care training*				
No	279	258	1	1
Yes	10	6	1.54 (0.55, 4.30)	1.43 (0.49, 4.14)

^*∗∗*^ *P* < 0.05.

## Data Availability

All necessary data are included within the manuscript.
